# Raddeanin A Induces Apoptosis and Cycle Arrest in Human HCT116 Cells through PI3K/AKT Pathway Regulation In Vitro and In Vivo

**DOI:** 10.1155/2019/7457105

**Published:** 2019-05-26

**Authors:** Chunqin Meng, Yuhao Teng, Xiaodong Jiang

**Affiliations:** ^1^Department of Chinese and Western Medicine, Nanjing Jiangning Hospital, The Affiliated Jiangning Hospital of Nanjing Medical University, Nanjing 211100, Jiangsu, China; ^2^Department of Oncology, Jiangsu Province Hospital of Traditional Chinese Medicine, Nanjing 210029, Jiangsu, China; ^3^Department of Oncology, Lianyungang First People^'^s Hospital, Lianyungang 222002, Jiangsu, China

## Abstract

This study aimed to investigate the in vitro and in vivo effects of Raddeanin A on apoptosis and the cell cycle in the human colorectal cell line, HCT116, and to explore the possible underlying mechanisms of action. We found the growth inhibition rate gradually increased as the drug concentration increased via the 3-(4,5)-dimethylthiahiazo(-z-y1)-3,5-di-phenytetrazoliumromide (MTT) assay, which indicated that Raddeanin A significantly inhibited the growth of HCT116 cells. Flow cytometry (FCM) showed that Raddeanin A concentration-dependently induced apoptosis in HCT116 cells. In addition, the percentage of cells in the G_0_/G_1_ phase was noticeably increased, which indicated that Raddeanin A blocked cell cycle progression in HCT116 cells and caused arrest in the G_0_/G_1_ phase. Moreover, the expression of proteins involved in the PI3K/AKT signaling pathway (e.g., p-PI3K and p-AKT) was decreased. The results showed that in vivo revealed that Raddeanin A significantly inhibited tumor growth in an HCT116-xenografted mouse model; apoptotic cells were also detected in the tumor tissue. The expression of the tissue proteins cyclinD1, cyclinE, p-PI3K, and p-AKT was decreased. The above results show that the Raddeanin A exerted a strong antitumor effect in the human colorectal cell line HCT116 both in vitro and in vivo. This effect may be caused by the induction of apoptosis and cycle arrest achieved through PI3K/AKT signaling pathway regulation.

## 1. Introduction

The PI3K/AKT pathway, which is prevalent in a variety of cell signal transduction pathways, is one of the current “hot topics” in cancer research. Studies have found that the pathway plays an important role in the physiological activity of cancer cells, such as cell energy metabolism, cell proliferation, invasion ability, cell apoptosis, and the cell cycle [1, 2]. The main members of this pathway are PI3K, AKT, and mTOR. Under the regulation of upstream factors, PI3K phosphorylates PIP2 to PIP3, and PIP3 promotes the binding of AKT to the cell membrane; PDK phosphorylates AKT to p-AKT, which indirectly activates mTOR. mTOR subsequently phosphorylated and activates downstream p70s6k and other factors, to control cell translation processes, such as ribosome biosynthesis, metabolism, and other important physiological functions, especially the inhibition of apoptosis and cell cycle [3]. Based on the role of the PI3K/AKT pathway in cancer development, some inhibitors of cell signaling molecules have been increasingly used for the clinical treatment of tumors, such as wortmannin and LY294002. Experimental results have shown that by inhibiting the pathway the vitality of tumor cells was reduced and the sensitivity of cells was increased to chemotherapy and radiotherapy [4, 5]. Based on the results that the effects of multiple antitumor were achieved by inhibiting the key proteins in the PI3K/AKT signaling pathway, a new trend is expected that will combine the traditional antitumor drugs with molecular targeted drugs; this would not only enhance the antitumor effect, but also reduce the adverse reactions of the drugs [6].

Raddeanin A (RA) is an oleanane-type triterpenoid saponin extracted from the herb* Anemone raddeana* Regel. Owing to its significant antitumor activity [7, 8], it has been extensively investigated. Studies have shown that RA can suppress the growth of a wide variety of tumor cells in vitro, such as liver cancer [9], breast cancer [10], gastric cancer [11], and ovarian cancer cells [12]. However, there are few studies on the effect of the compound on colorectal cancer cells, especially with regard to in vivo antitumor activity. The present study was designed to investigate the antitumor effect of RA in the colorectal cancer line HCT116 both in vitro and in vivo. We also investigated the possible underlying mechanisms of these effects.

## 2. Materials and Methods

### 2.1. Reagents and Antibodies

RPMI-1640 medium and fetal bovine serum (FBS) were acquired from Gibco BRL (Gaithersburg, MD, USA). RA was purchased from the China National Institute for the Control of Pharmaceuticals, dissolved in dimethyl sulfoxide (DMSO), and stored at −20°C. MTT, TUNEL staining kit, and LY294002 were obtained from Sigma Chemical Company (St. Louis, MO, USA). Annexin-V/Propidium Iodide (PI) apoptosis detection kits were obtained from BD Biosciences (Franklin Lakes, NJ, USA). Primescript reverse transcription reagent kits with gDNA erasers were obtained from TaKaRa (Dalian, China). TRIzol reagent and Power SYBR Green PCR Master Mixes were purchased from Life Technologies (Grand Island, NY, USA). The primary antibodies against BAX, Bcl-2, PARP, caspase-3, cleaved caspase-3, cyclinD1, CDK2, PI3K, AKT, p-PI3K, p-AKT, and *β*-actin were obtained from Cell Signaling Technology (Beverly, MA, USA). Fluorescein-conjugated secondary antibodies were obtained from Odyssey (Licor, Belfast, ME, USA). The mice were obtained from Kevins Animal Company (Changzhou, China) and maintained in a specific-pathogen-free (SPF) environment with adequate food and water.

### 2.2. Cell Lines and Culture

The human colorectal cancer cell line HCT116 was provided by the Shanghai Institute for Biological Research (Shanghai, China) and cultured in RPMI-1640 medium supplemented with 10% FBS in a humidified atmosphere with 5% CO_2_ at 37°C.

### 2.3. MTT Assay

Cells in the logarithmic growth phase were plated in 96-well culture plates. After treatment with RA or DMSO for 12h, MTT was added to the cells (5 mg/mL) and incubated for 4 h. DMSO was then added to dissolve the reaction products and the optical densities (ODs) were measured at 490 nm using an ELx800 microplate reader (BioTek, Winooski, VT, USA). The inhibition rate was calculated from the following equation:

(1) $$\begin{array}{c} Inhibition\;rate=\left(\;1–\frac{{OD}_{experiment}}{{OD}_{control}}\;\right)\times 100\%. \end{array}$$

### 2.4. Flow Cytometric Analysis

Apoptosis test: the cells were detached by trypsinization, treated with RA for 12 h, washed twice with PBS, and resuspended in 500 *μ*L binding buffer containing 5 *μ*L annexin-V-FITC and 5 *μ*L PI. Prior to flow cytometric analysis, the cells were incubated for 15 min in the dark.

Cell cycle test: the cells were treated with RA for 12 h, washed with cold PBS twice, and fixed with 70% ethanol for 12 h. The cells were prepared with the cycle kits in accordance with the manufacturer's instructions. After the addition of buffer, RNase A, and PI, the cells were incubated for 30 min in the dark at 37°C and then analyzed by flow cytometry.

### 2.5. Reverse Transcription-Polymerase Chain Reaction (RT-PCR) Assay

The tumor tissue was lysed and the tissue RNA and cell RNA was extracted from tumor tissue and cells separately in accordance with the instructions for TRIzol reagent. After the purity of the RNA was spectrophotometrically confirmed, the RNA was reverse-transcribed into cDNA, using the TaKaRa RT retrovirus kit. The PCR amplification reaction and data analysis were performed on an ABI 7500 fast RT-PCR System. The 2^-∆∆Ct^ method was followed to analyze the results in accordance with the following equations:

(2) $$\begin{array}{c} ∆Ct={Ct}_{target\;genes}–{Ct}_{endogenous\;reference\;gene} \end{array}$$

and

(3) $$\begin{array}{c} ∆∆Ct=∆{Ct}_{treated\;samples}–∆{Ct}_{control\;samples} \end{array}$$

in which *β*-actin was used as the reference gene. The final gene expression level was reported in the form 2^-∆∆Ct^. The gene primers were designed by Primer Express and are shown in Table 1.

### 2.6. Western Blot Analysis(WB)

After treatment with RA, the cells and tumor tissue were lysed in RIPA to obtain proteins. The protein concentration was assessed by the Bradford method (BCA). The expression of *β*-actin protein was used as a loading control. For each sample, an equal volume of protein was loaded into 10% or 12% SDS-polyacrylamide gel for electrophoresis and transferred by electroblotting to polyvinylidene difluoride (PVDF) membranes (Millipore, Boston, MA, USA). The PVDF membrane was blocked by incubation in 5% BSA for 1 h and then incubated with antibodies against BAX (1:1000 dilution), Bcl-2 (1:1000 dilution), caspase-3 (1:1000 dilution), cleaved caspase-3 (1:1000 dilution), PARP (1:1000 dilution), cleaved PARP (1:1000 dilution), PI3K (1:1000 dilution), AKT (1:1000 dilution), p-PI3K (1:1000 dilution), p-AKT (1:1000 dilution), cyclinD1 (1:1000 dilution), cyclinE (1:1000 dilution), CDK4 (1:1000 dilution), and CDK2 (1:1000 dilution) overnight. The membrane should be washed by Tris Buffered Saline with Tween-20 (TBST) three times (5 min/time) and then incubated with the appropriate secondary fluorescent antibody at a 1:3000 dilution. Finally, the signal intensity of the membranes was examined by an Odyssey analyzer (LICOR, Belfast, ME, USA).

### 2.7. Xenograft Tumor Model

HCT116 cells in the logarithmic growth phase were harvested and injected into 5-week-old BALB/c nude mice (equal numbers of male and female mice; 1 × 10^6^ cells/mouse) to establish the xenograft tumor model. The mice were randomly divided into two groups (three mice per group) and when the tumors reached approximately 150 mm^3^, the control group was injected with PBS (100 *μ*L) and the experimental group was injected with RA (4 mg/kg). The tumor volume and body weight were recorded every other day and the tumor volume was calculated from the following formula: volume (mm^3^) = (length × width^2^)/2. After 2 weeks, the tumor tissue was removed from the mice. The tumor volume and weight were measured and used to calculate the inhibitory rate from the following equation:

(4) Inhibitory rate IR,%=volumeweightcontrol group–volumeweightexperimental groupvolumeweightcontrol group×100%.

### 2.8. Hematoxylin-Eosin (HE) Staining

Tumor tissue and liver tissue were fixed with 4% paraformaldehyde, embedded in paraffin for 10 min, sliced, and processed for routine HE staining. After washed with dimethylbenzene, the tissue was mounted with neutral balsam. All slices were photographed by a Leica DFC 320 photomicroscope.

### 2.9. TdT-Mediated dUTP Nick-End Labeling (TUNEL) Assay

To analyze the apoptotic cells in the xenograft tumor, the tumor tissue was fixed in 4% paraformaldehyde overnight, embedded in paraffin, and sliced. The slices were sequentially rinsed with different concentrations of ethanol. In accordance with the directions of TUNEL kit, the tissue slice was incubated with the compound reaction mixture for 60 min at 37°C in the dark and the cell nucleus was stained with DAPI. For each tissue slice, three random fields of vision were photographed. The apoptosis rate was calculated from the following formula by Image-Pro Plus 6.0 software: Apoptosis rate=Number of positive cells/Total number of cells × 100%.

### 2.10. Immunohistochemistry

Immunohistochemical staining was performed to determine the expression of autophagy- and apoptosis-related proteins. First, the tumor tissue was fixed in 4% paraformaldehyde, embedded in paraffin, and sliced into sections, which were then deparaffinized. The endogenous peroxidase activity was destroyed by incubation with 3% H_2_O_2_. Second, after nonspecific antigenic sites were blocked by incubation with 3% BSA for 30 min at room temperature, the tissues were incubated with primary antibodies overnight at 4°C and then with the appropriate secondary antibody. Finally, the tissues were counterstained with hematoxylin and observed under a fluorescence microscope.

### 2.11. Statistical Analysis

The software SPSS16.0 was used to compute the statistical analyses. The measurements are presented as the mean ± standard deviation (SD). The difference between groups was examined by one-way ANOVA followed by Dunnett's test, with P<0.05 considered to indicate statistically significant data.

## 3. Results

### 3.1. Effect of RA on HCT116 Cell Growth In Vitro

**Figure 1:**
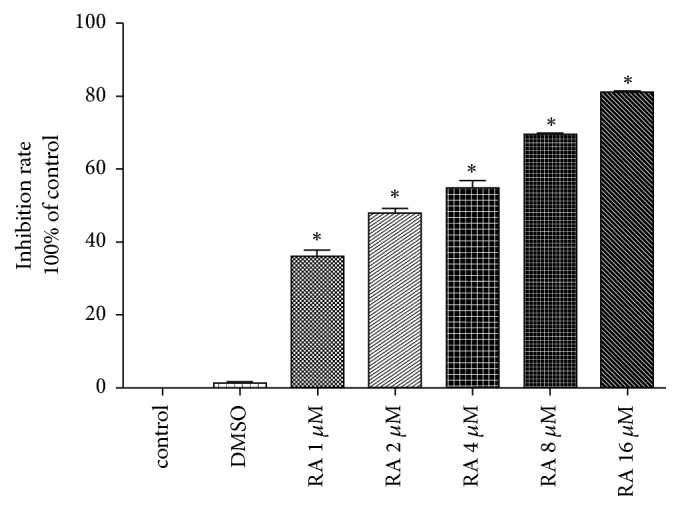
Raddeanin A (RA) inhibits HCT116 cell proliferation. The MTT assay showed that cellular viability was positively correlated with RA concentration (1, 2, 4, 8, or 16 *µ*M).

The MTT assay indicated that RA significantly inhibited the proliferation of HCT116 cells. As the drug concentration increased (1, 2, 4, 8, and 16 *μ*M), a gradual increase in the inhibition rate was observed. Even at lower concentrations of RA (1 *μ*M), the inhibition rate was (35.1±0.8)%, when 16 *μ*M RA in cells, the inhibition rate as high as (81.2±1.2)%, which demonstrated that RA had a good inhibition in HCT116 cells, whereas DMSO had little effect on cell viability. The IC50 of 12 h was 2.61 *μ*M (Figure 1).

### 3.2. Effect of RA on HCT116 Cell Apoptosis and Cell Cycle

**Figure 2:**
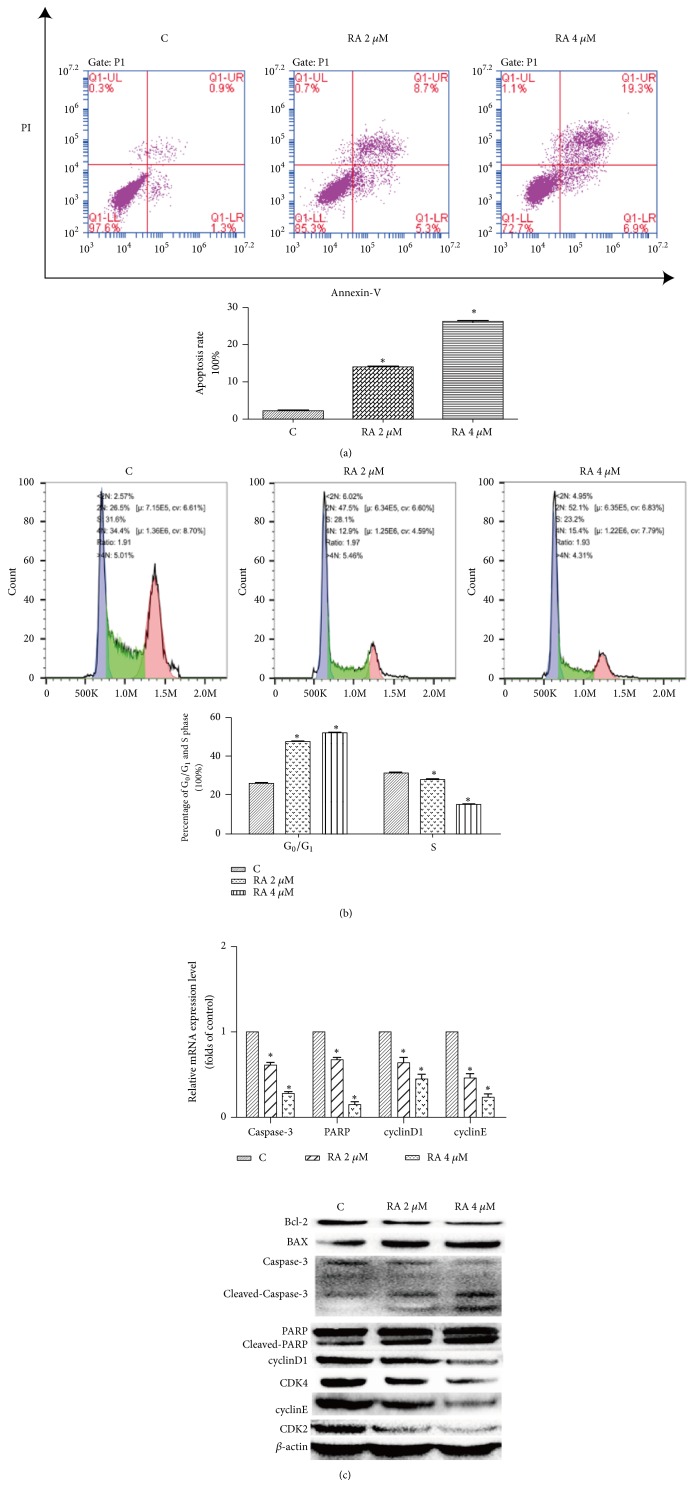
Raddeanin A (RA) induced apoptosis and blocked cell cycle progression in HCT116 cells. (a) After treatment with RA (2 and 4 *µ*M) for 12 h, the cells were incubated with annexin-V-FITC and propidium iodide (PI) and the apoptosis rate was analyzed via flow cytometry. The results shown are from a representative experiment. (b) Representative results of cell cycle analysis conducted by flow cytometry in HCT116 cell treated with different doses of RA for 12 h (n = 3). (c) The expression of apoptotic proteins and cell cycle-related factors was detected by RT-PCR and western blotting. The data shown are the mean ± SD (n=3, *∗*P<0.05, compared with the control). *β*-actin was used as an internal control.

As the MTT results indicated that RA exerted a marked antitumor effect, we attempted to identify the mechanism of this effect. It is well known that indefinite proliferation is a predominant characteristic of tumor cells; studies have indicated that the main reason for this is an absence of apoptosis mechanisms and cell cycle disorder. Thus, we examined the apoptosis and cell cycle of the treated cells. The apoptosis assay indicated that RA induced apoptosis in HCT116 cells. Compared with the control group (2.2±1.1)%, the treatment of HCT116 cells with 2 *μ*M RA resulted in an apoptosis rate of (14.0±0.8)%, which increased to (26.2±0.3)% with 4 *μ*M RA (P <0.05), the difference was statistically significant (Figure 2(a)). In the cell cycle assay, the percentage of G_0_/G_1_ phase and S phase cells in the control group was (26.5±1.2)% and (31.6±1.8)%, respectively, whereas the percentage of G_0_/G_1_ cells in the groups treated with the test compound increased to (47.5±1.3)% (2 *μ*m RA) and (52.1±2.1)% (4 *μ*M RA) and the percentage of S phase cells was simultaneously decreased to (28.1±0.7)% (2 *μ*M RA) and 23.2±0.9 (4 *μ*M RA), which indicated 6that RA could block the cells in G_0_/G_1_ phase (Figure 2(b)). Furthermore, WB results showed that the expression of apoptotic proteins, such as cleaved caspase-3, cleaved PARP, and BAX, increased, whereas that of caspase-3, PARP, and Bcl-2 decreased. The expression of cell cycle-related factors cyclinD1, cyclinE, CDK4, and CDK2 was also decreased (Figure 2(c)).

### 3.3. Effect of RA on PI3K/AKT Signaling Pathway

**Figure 3:**
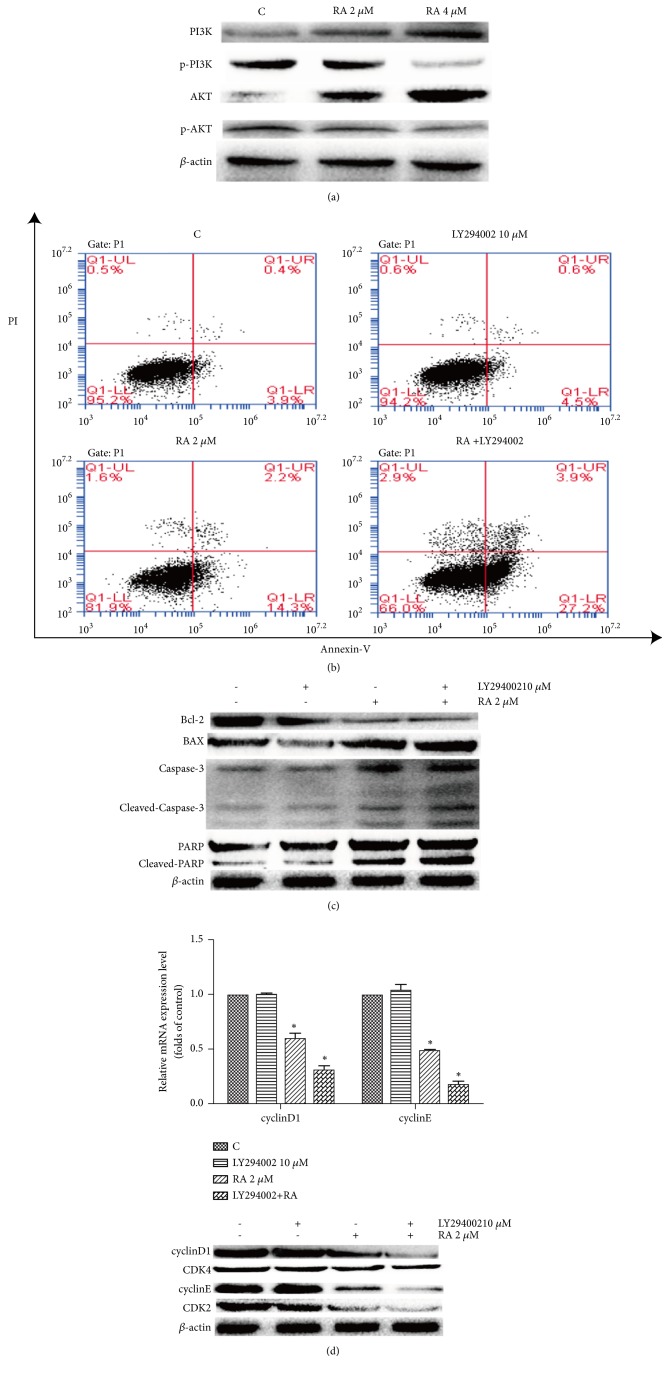
The PI3K/AKT signaling pathway was involved in the regulation of apoptosis and the cell cycle by Raddeanin A RA. (a) Immunoblot assay for the PI3K-AKT pathway. (b) Prior to treatment with RA (2 *µ*M), the cells were treated with LY294002 (a PI3K inhibitor; 10 *µ*M) for 1 h. The cells were incubated with annexin-V-FITC and propidium iodide (PI) and the apoptosis rate was analyzed via flow cytometry. (c) Protein levels of apoptotic proteins were determined via western blotting. *β*-actin was used as an internal control. (d) The mRNA and protein levels of cell cycle proteins were determined via RT-PCR and western blotting, respectively. *β*-actin was used as an internal control. The data are presented as the mean ± SD (n=3, *∗*P<0.05, compared with the control).

The physiological activities of the cell, together with apoptosis and the cell cycle, are regulated by signaling sequences. In this study, WB analysis revealed that p-PI3K and p-AKT proteins were downregulated, whereas PI3K and AKT were upregulated (Figure 3(a)). This result showed that the PI3K/AKT signaling pathway was suppressed. To confirm that the PI3K/AKT signaling pathway was involved in the regulation of cell apoptosis and cell cycle by RA, the PI3K inhibitor LY294002 was employed. The flow cytometry results indicated apoptosis rates of (16.5±1.1)% in the cells treated with RA (2 *μ*M) and (31.1±1.2)% in the cells treated with RA (2 *μ*M) and LY294002 (10 *μ*M) together (Figure 3(b)). Furthermore, the expression of cleaved caspase-3, cleaved PARP, and BAX was also increased compared with that in the RA-treated cells, while caspase-3, PARP, and Bcl-2 decreased (Figure 3(c)). With regard to the cell cycle, the RT-PCR and WB analyses showed that the expression of cyclinD1, cyclinE CDK4, and CDK2 decreased after HCT116 cells were treated with LY294002 and RA (Figure 3(d)).

### 3.4. Effect of RA on Mouse Xenograft Model

**Figure 4:**
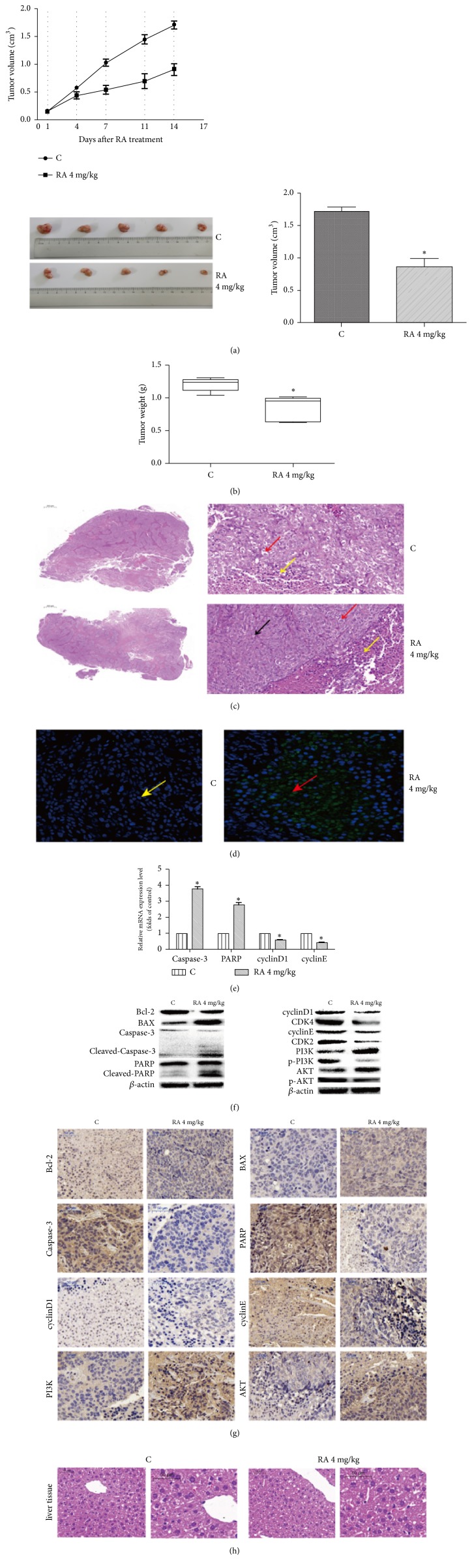
Raddeanin A- (RA-) induced apoptosis and cell cycle arrest might occur through the regulation of the PI3K/AKT the pathway in vivo. (a,b) After treatment, the statistical analysis of tumor volume and tumor weight was conducted. The data shown are the mean ± SD (n=3, *∗*P<0.05, compared with the control). (c) The necrosis of tumor tissue was detected by HE staining. Yellow arrow, inflammatory cells; black arrow, pathologic mitosis; red arrow, vacuolated cytoplasm. (d) Apoptosis cell of tumor tissue was detected by TUNEL staining. Apoptotic cells are indicated by red arrow, yellow arrow indicated normal cells (20x). (e, f, g) RT-PCR, immunoblot assay, and immunohistochemistry indicate the expression of apoptosis-related factors, cell cycle-related factors, and PI3K/AKT pathway factors. (h) HE staining tests the effect of RA on liver. *β*-actin was used as the internal control. The data are shown as the mean ± SD (n=3, *∗*P<0.05, compared with the control).

To investigate the antitumor effect of RA in vivo, we selected a colorectal cancer HCT116 cell line to build a nude mouse xenograft model. As shown in Figures 4(a) and 4(b), the average tumor volume and weight in the control group were (2.01±0.86) cm^3^ and (2.31±1.36) g, respectively, and (1.01±1.36) cm^3^ and (1.26±1.05) g in the RA-treated group (p <0.05). To further explore the underlying in vivo mechanism, we conducted HE staining, TUNEL assay, immunohistochemistry, and WB analysis. The HE staining of the tumor tissue showed that the necrotic area was less than one-quarter of that in the control group and the necrotic area of the treatment group was almost half (Figure 4(c)). TUNEL staining showed the presence of apoptotic cells in the RA group with an apoptosis rate of (43.6±1.26)%, whereas there were few apoptotic cells in the control group (Figure 4(d)). WB analysis indicated that the tissue proteins of cleaved caspase-3, cleaved PARP, and BAX were more highly expressed than in the control group, while caspase-3, PARP and Bcl-2 were lower expression; immunohistochemistry also indicated a low expression of caspase-3, PARP, and Bcl-2 except BAX (Figures 4(e), 4(f), and 4(g)). Additionally, we not only found that expression of cell cycle-related proteins cyclinD1, cyclinE, CDK4, and CDK2 (Figures 4(e), 4(f) and 4(g)) were decreased but also confirmed the downregulation of p-PI3K and p-AKT proteins and upregulation of PI3K and AKT proteins through WB and immunohistochemistry (Figures 4(f) and 4(g)). These results demonstrated that RA-induced apoptosis and arrest of the tumor cells might occur through regulation of the PI3K/AKT pathway in vivo. We also investigated the possible side effects of RA in vivo. HE staining revealed that there were no structural differences between the control and RA-treated groups (Figure 4(h)).

## 4. Discussion

In recent years, with the development of traditional Chinese medicine, more traditional Chinese medicines were used to treat tumor and were proved to be effective. They can inhibit tumor growth through various mechanisms, such as by inducing apoptosis and autophagy, blocking the cell cycle and regulating signal pathways, and so on. Apoptosis (PCD I), which is regulated by a series of signals, is a programmed cell death mechanism. The cell morphological and biochemical features in apoptosis are DNA rupture, nuclear pyknosis, karyorrhexis, and karyolysis, accompanied by the characteristic formation of apoptosis bodies and caspase activation, which finally leads to the consumption of the cell by lysosomes [13]. The absence of apoptotic mechanisms is one of the characteristic features of a tumor; thus, the induction of apoptosis has emerged as a main method for treatment of tumors. Caspase-3 is one of the most important apoptosis executors of the caspase family and is also the main effect factors in the process of cell apoptosis; cleaved caspase-3 is considered to be a sign of apoptosis. In this study, we found that the expression of cleaved caspase-3 was upregulated, which indicated that RA could induce apoptosis in HCT116 cells both in vitro and in vivo. In addition to the lack of apoptotic mechanism, disorder of the cell cycle is also a feature of malignant tumors [14]. The cell cycle is one of the most important processes in the cell life. G_1_ phase is the key to the start of the cell cycle in all phases, while cyclin D and CDK4 play an important role in G_1_ phase which can start the cell cycle and promote the synthesis of DNA. Our experiments indicated that RA could block the cell cycle in the G_0_/G_1_ phase to inhibit cellular proliferation. However, for the regulation of apoptosis and the cell cycle, research has focused on the PI3K/AKT signal pathway. Studies have found that the PI3K/AKT signaling pathway can control expression of Bad [15], caspase-9, Par 4 [16], p53 [17], and other factors, which prevents the mitochondrial apoptosis [18]. In addition, it can also activate the protein kinases of the cell cycle, increase the expression of cell cycle proteins, and promote cell cycle progression, cell proliferation, and differentiation, which subsequently promote tumor development [19]. In this study, we found the expression of p-PI3K and p-AKT downregulated, which suggested that the PI3K/AKT pathway might be involved in the regulation of apoptosis and the cycle. By blocking this pathway with LY294002, we confirmed this speculation. To confirm the antitumor effect of RA in vivo, a nude mouse xenograft model was employed. And we found RA could effectively inhibited tumor growth in vivo. We also explored the underlying mechanisms of the effect of RA. Through HE staining, TUNEL staining, RT-PCR, and WB assays, we found that RA could induce apoptosis and block the cell cycle to inhibit tumor growth by regulating PI3K/AKT pathway in vivo which is consistent with the experimental results in vitro. What is more important, given the significant antitumor effect of RA, we also found RA was not a toxic drug.

In conclusion, RA can effectively inhibit the growth of the colorectal cancer cell line HCT116 both in vitro and in vivo; this antitumor efficiency is likely to be achieved through the induction of apoptosis and cycle arrest, which occurs via regulation of the PI3K/AKT pathway. Furthermore, RA exerts no toxicity in the liver. These findings suggest that RA is a promising candidate for the treatment of colorectal cancer and lay the foundation for further clinical applications of this compound.

## Figures and Tables

**Table 1:** Primer sequences used in the reverse transcription-polymerase chain reaction (RT-PCR) amplifications.

Gene primer	Sequence (5′-3′)	Length of PCR product (bp)
Caspase-3	F: TGGACCTGTTGACCTGA	269
	R:CACAAAGCGACTGGATG	
PARP	F:ACGCACAATGCCTATGAC	168
	R:CCAGCGGAACCTCTACAC	
cyclinD1	F:TTGCTGCTATTGGAGGATCAGT	204
	R:TGGCTAAGTGAAGCATGAGGTA	
cyclinE	F:TTTGCAGGATCCAGATGAAGA	187
	R:CACAGACTGCATTATTGTCCCAAG	
*β*-Actin	F: GGCCAACCGCGAGAAGAT	134
	R: CGTCACCGGAGTCCATCA	

## Data Availability

The data used to support the findings of this study are available from the corresponding author upon request.
